# Xeroderma pigmentosum group C sensor: unprecedented recognition strategy and tight spatiotemporal regulation

**DOI:** 10.1007/s00018-015-2075-z

**Published:** 2015-10-31

**Authors:** Marjo-Riitta Puumalainen, Peter Rüthemann, Jun-Hyun Min, Hanspeter Naegeli

**Affiliations:** Institute of Pharmacology and Toxicology, University of Zürich-Vetsuisse, 8057, Zurich, Switzerland; Science for Life Laboratory, Division of Translational Medicine and Chemical Biology, Department of Medical Biochemistry and Biophysics, Karolinska Institute, Stockholm, Sweden; Department of Chemistry, University of Illinois at Chicago, Chicago, IL 60607 USA

**Keywords:** Aging, Diurnal life, DNA repair, Genomic instability, Skin cancer, SUMO, Sunburn, Tumor suppressor, Ubiquitin

## Abstract

The cellular defense system known as global-genome nucleotide excision repair (GG-NER) safeguards genome stability by eliminating a plethora of structurally unrelated DNA adducts inflicted by chemical carcinogens, ultraviolet (UV) radiation or endogenous metabolic by-products. Xeroderma pigmentosum group C (XPC) protein provides the promiscuous damage sensor that initiates this versatile NER reaction through the sequential recruitment of DNA helicases and endonucleases, which in turn recognize and excise insulting base adducts. As a DNA damage sensor, XPC protein is very unique in that it (a) displays an extremely wide substrate range, (b) localizes DNA lesions by an entirely indirect readout strategy, (c) recruits not only NER factors but also multiple repair players, (d) interacts avidly with undamaged DNA, (e) also interrogates nucleosome-wrapped DNA irrespective of chromatin compaction and (f) additionally functions beyond repair as a co-activator of RNA polymerase II-mediated transcription. Many recent reports highlighted the complexity of a post-translational circuit that uses polypeptide modifiers to regulate the spatiotemporal activity of this multiuse sensor during the UV damage response in human skin. A newly emerging concept is that stringent regulation of the diverse XPC functions is needed to prioritize DNA repair while avoiding the futile processing of undamaged genes or silent genomic sequences.

## Introduction

Living organisms are relentlessly challenged by exogenous and endogenous DNA-damaging agents that threaten genome integrity. Prominent types of DNA damage are “bulky” lesions consisting of base adducts or intrastrand crosslinks that destabilize complementary base pairing in the double helix. Such base pair-disrupting injuries arise from chemical carcinogens such as polycyclic aromatic hydrocarbons forming covalent base adducts [1], reactive drugs like cisplatin generating crosslinks between adjacent bases [2] or by-products of cellular metabolism including oxygen radicals yielding cyclodeoxynucleosides [3, 4]. The most commonplace lesions derive from exposure to the ultraviolet (UV) range of natural sunlight or artificial radiation sources, which induce crosslinks between neighboring pyrimidines, i.e., cyclobutane pyrimidine dimers (CPDs) and (6-4) photoproducts (6-4PPs) [5]. If not promptly removed by DNA repair, these UV crosslinks like other bulky lesions interfere with transcription [6], DNA replication or cell cycle [7], and cause mutations and chromosomal aberrations that culminate in cancer as well as accelerated aging (reviewed by [8]). In particular, the incidence of skin cancer continues to increase, and thus remains a public health concern, despite widespread awareness that sunlight is the major risk factor for cutaneous malignancies [9, 10]. This review is focused on recent advances in our knowledge of how XPC protein carries out its DNA quality surveillance preventing sunlight-induced skin cancer. Since the discovery that DNA repair of UV damage critically depends on post-translational protein modifications [11, 12], it has become increasingly clear that multiple polypeptide modifiers control the pleiotropic activity of this versatile sensor of DNA integrity.

## Excision repair of bulky DNA lesions

Nucleotide excision repair (NER) is the DNA repair system that removes bulky base lesions induced by chemical carcinogens, DNA-reactive drugs, by-products of aerobic metabolism or UV light. Being caused by various DNA-damaging agents, these NER substrates are structurally diverse, but always limited to one DNA strand. The cut-and-patch NER machinery operates by cleaving this damaged strand on either side of the injury, thereby excising the lesion as part of 24–32-nucleotide-long single-stranded segments [13, 14].

Depending on the context of its occurrence, DNA damage is detected by two alternative routes. In the transcription-coupled sub-pathway (TC-NER), damage is first sensed when the RNA polymerase II complex encounters obstructing base lesions during transcription [15]. This molecular collision with roadblocks triggers a reaction that is not yet fully understood, but eventually promotes the accelerated removal of base lesions from the transcribed strand of active genes (reviewed by [8, 16, 17]). On the other hand, bulky DNA lesions anywhere in the genome are detected, independently of RNA polymerase II, by a more general sub-pathway known as global-genome NER (GG-NER; reviewed by [18]). Defects in GG-NER result in the xeroderma pigmentosum (XP) syndrome, a devastating cancer-prone condition characterized by photosensitivity, severe sunburns and freckling, solar keratosis and an over 1,000-fold increased risk of sunlight-induced skin cancer [19]. XP patients also have a higher propensity of developing internal tumors attributable to chemical carcinogens or reactive oxygen species [20]. These patients are classified into different genetic complementation groups (from XPA to XPG) caused by mutations in the respective seven NER genes [21]. An eighth complementation group (XP-V) presents a variant form resulting from mutations in the gene coding for DNA polymerase η, which is responsible for the error-free bypass of UV lesions during DNA replication [22].

## Core GG-NER machinery

A key feature of the GG-NER pathway is that it takes only a limited set of proteins to recognize and repair an extraordinarily wide spectrum of bulky lesions. Inducing tiny spots of UV damage in cell nuclei by irradiation through micropore filters is a frequently adopted strategy to study the intracellular trafficking of these GG-NER factors. In combination with biochemical reconstitution assays, this method demonstrated that locating bulky lesions depends on a heterotrimeric factor composed of xeroderma pigmentosum group C (XPC; [23, 24]) one of two human RAD23 homologs (predominantly RAD23B; [25]) and centrin 2 (CETN2; [26–28]). The DNA-binding and lesion recognition activity of this heterotrimeric complex resides entirely within the XPC subunit. The contribution of RAD23B (a 26S proteasome-interacting factor that escapes proteolytic degradation) and CETN2 (a calcium-binding protein also found in centrosomes) is to protect XPC from degradation and support its proper folding necessary to achieve optimal DNA-binding affinity [25, 29, 30]. The RAD23B partner supports the recognition of damaged DNA by XPC protein [31, 32] but is readily released once XPC associates with DNA lesions [32, 33]. On the other hand, CETN2 may remain associated with target sites, while still in complex with XPC, and facilitate downstream recognition steps [34].

**Fig. 1:**
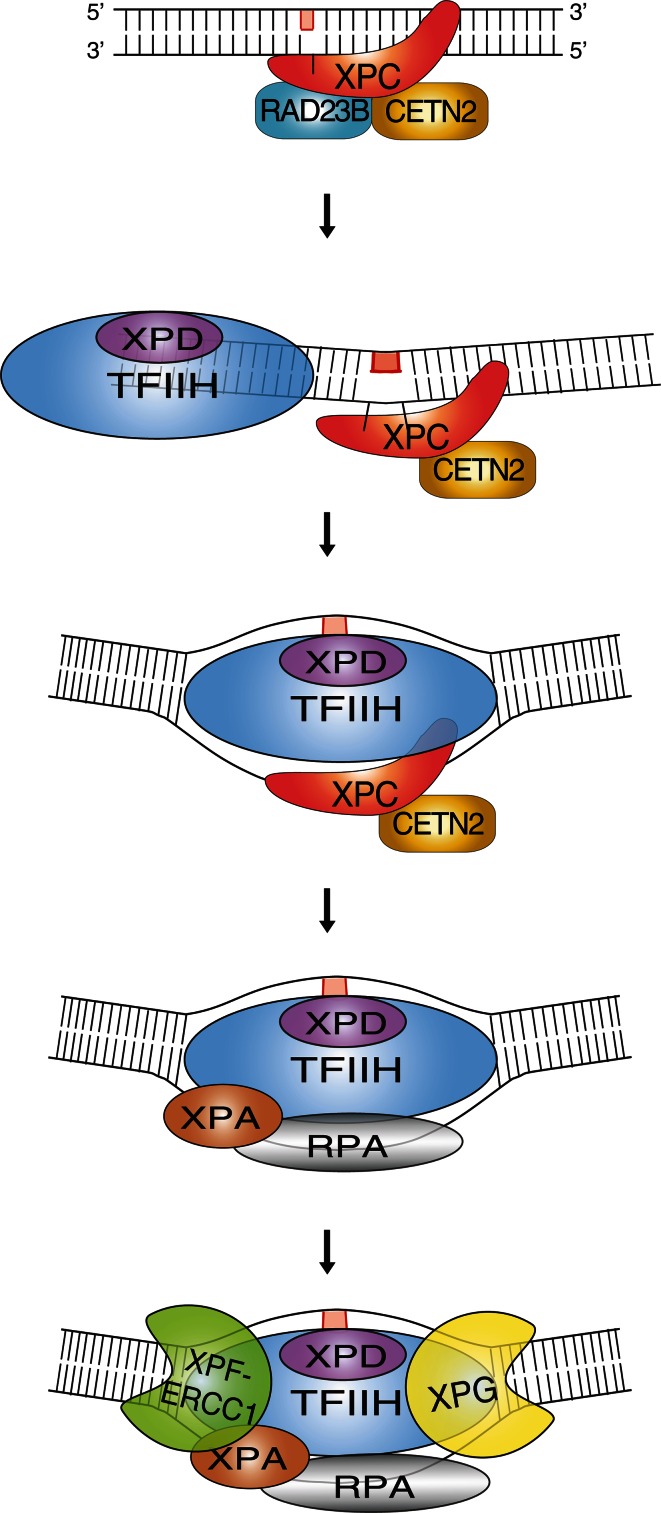
Initiation of GG-NER activity by the heterotrimeric XPC complex. The XPC subunit is a thermodynamic sensor that recognizes base pair destabilizations of the DNA double helix caused by bulky lesions such as UV light-induced 6-4PPs or carcinogen-DNA adducts (symbolized by the *red rectangle* in the upper damaged strand). Briefly, the GG-NER reaction proceeds by a stepwise mechanism initiated by the trimeric XPC-RAD23B-CETN2 complex, which binds to ruptured base pairs and extends the local melting of DNA by flipping-out two nucleotides of the undamaged strand opposite to bulky lesions. After this initial sensing of damaged sites, the XPC subunit mediates the recruitment of XPD as part of the multimeric TFIIH complex. The DNA helicase activity of XPD is exploited to scan the damaged strand and, after reaching the injured base, this tracking enzyme forms a long-lived demarcation complex with the DNA duplex being unwound around the lesion. The single-stranded configuration of DNA in this intermediate is stabilized by RPA, which together with XPA positions the two structure-specific endonuclease “scissors” (XPF-ERCC1 and XPG) in a way that they cut the damaged strand at each Y-shaped double-stranded to single-stranded DNA junction. This dual cleavage results in the removal of bulky lesions in the form of oligonucleotide segments with a length of 24–32 residues. For the special case of CPDs, this GG-NER system needs the assistance of the DDB2 damage detector for substrate recognition

The XPC-CETN2 heterodimer bound to DNA substrates forms a recruiting platform for transcription factor IIH (TFIIH; Fig. 1). This 10-subunit complex comprises the XPD helicase, which separates complementary strands in duplex DNA to generate an unwound configuration of about 25 nucleotides around the lesion [35, 36]. The resulting unwound intermediate is stabilized by XPA in conjunction with replication protein A (RPA), and the damaged strand is cut by structure-specific endonucleases at the double-stranded to single-stranded DNA junctions on each side of the lesion [35, 37]. Incision on the 5′ side is carried out by a heterodimer consisting of XPF and excision repair cross-complementing 1 (ERCC1), followed by the incision on the 3′ side through XPG [38]. Once the excised segment harboring the lesion is released, the resulting single-stranded gap is filled by DNA repair synthesis through the action of DNA polymerases *δ*, *ε* or *κ* [39]. Finally, full helix integrity is restored by DNA ligase I and DNA ligase IIIα that seal the nicks [40, 41].

## Molecular structure of the multi-domain XPC sensor

**Fig. 2:**
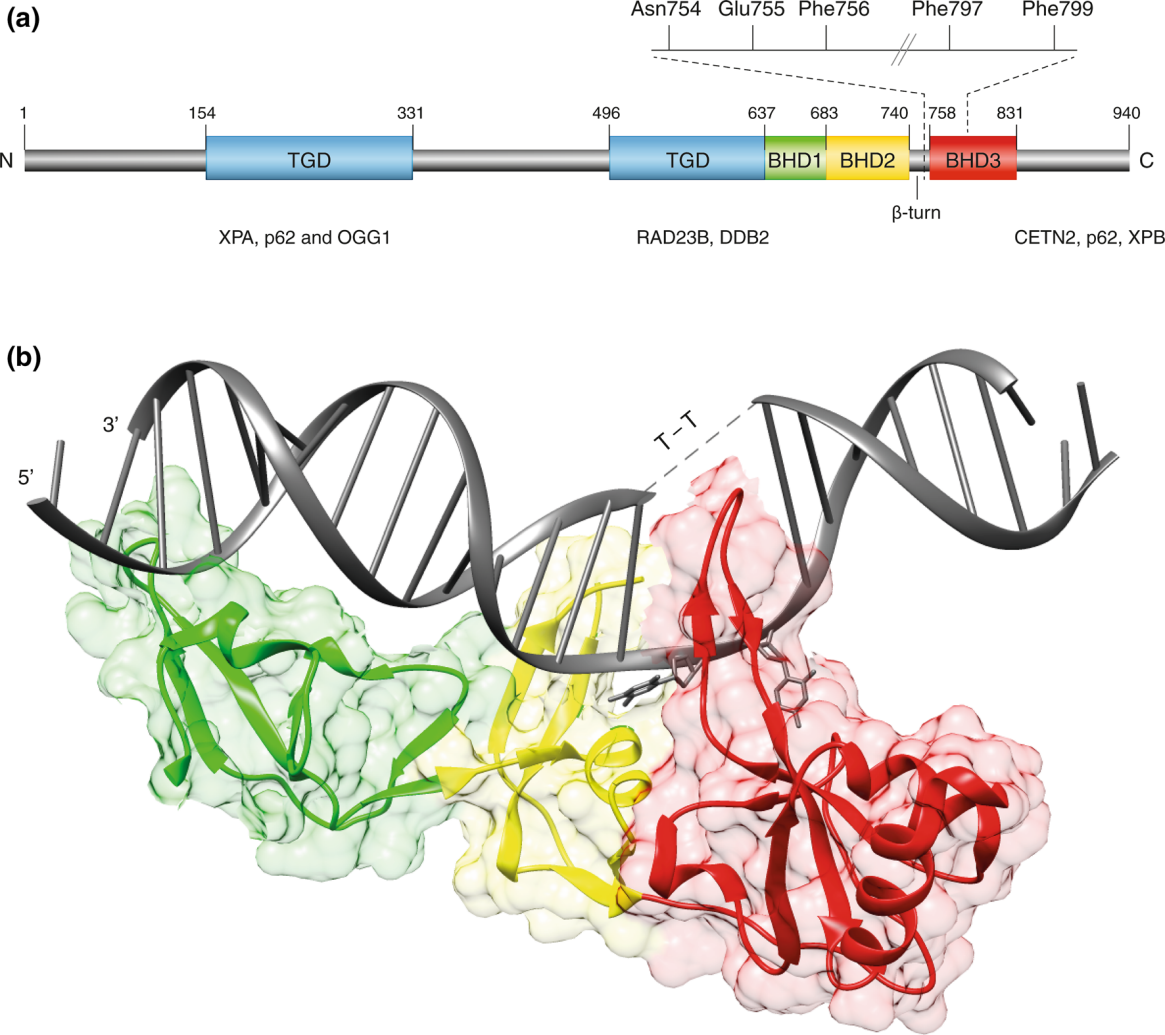
Modular structure of the XPC protein.** a** Domain map of human XPC protein highlighting the transglutaminase-like domain (TGD) and the three β-hairpin domains (BHD1-3) interacting with DNA. TGD and BHD1: in the crystal structure of the RAD4 homolog from *S. cerevisiae* [50], TGD region, in conjunction with BHD1, binds to 11 base pairs of double-stranded DNA flanking the lesion. BHD2 and β-turn subdomain: protein dynamics studies in human cells [62] indicate that BHD2 together with the β-turn detect unpaired bases in the damaged double helix. BHD3: XPC protein becomes anchored onto lesions sites by the intra-helical insertion of a long β-hairpin “finger” protruding from BHD3. DNA-attractive amino acids (Asn754, Phe756, Phe797, Phe799) and a DNA-repulsive residue (Glu755) are responsible for the sensing and flipping-out of unpaired bases in the undamaged strand opposite to bulky lesions. The aforementioned domains are also involved in interactions with various protein partners (XPA, p62, OGG1, RAD23B, DDB2, CETN2, and XPB).** b** Ribbon diagram of the BHD1-BHD3 region of RAD4 in complex with DNA containing a CPD (indicated by T–T) embedded in three consecutive base mismatches [50]. TGD region is not shown. *Green* BHD1; *yellow* BHD2; *red* BHD3; *gray* DNA. This structure reflects the stably damage-anchored protein. While the three mismatches were necessary to allow for the binding of RAD4 protein to DNA, there are no contacts between RAD4 and the two pyrimidines of the CPD lesion, which are disordered and concealed by the solvent. The figure was prepared with the Chimera extensible molecular modeling system, using the structure PDB 2QSG

The human *XPC* gene is located on chromosome 3, consists of 16 exons and codes for a protein of 940 amino acids [42]. The protein contains domains for binding to both DNA [43–45] and many protein partners (Fig. 2a). To serve as a common initiator of GG-NER activity, XPC protein must be able to sense a wide variety of chemically unrelated DNA lesions. As these different substrates share no chemical motif that would support a canonical “lock-and-key” recognition mechanism, it was a major challenge to understand how this promiscuous sensor inspects the DNA double helix for a broad lesion spectrum.

A first insight towards solving this substrate versatility enigma came from a comparison of amino acid sequences indicating that human XPC displays short regions of homology with single-stranded DNA-binding domains of RPA and breast cancer 2 (BRCA2). This homology suggested that XPC protein is able to detect the local single-stranded character of DNA containing base pair-disrupting lesions [46]. Biochemical experiments demonstrated that XPC indeed exhibits a binding preference for single-stranded oligonucleotides, or double-stranded DNA with single-stranded overhangs, over duplex counterparts [45, 47, 48]. It was also observed that DNA lesions induced by UV or cisplatin within single-stranded DNA reduce XPC binding, indicating that XPC protein may avoid contacting damaged nucleotides. This aversion for damaged nucleotides, together with the preference for single-stranded DNA elements, suggested an indirect sensing mechanism by which XPC protein recognizes unpaired nucleotides in the undamaged strand, thus exploiting a generic attribute of damaged DNA featuring compromised base pairing [46, 49].

This unique mode of action was confirmed when a high-resolution structure of the evolutionarily conserved homolog from *Saccharomyces cerevisiae* (Rad4 protein) came to light [50]. The co-crystal structure with duplex DNA containing a model lesion shows that Rad4 deploys four core domains that bind to damaged DNA in two parts (Fig. 2b). One part is made up of a transglutaminase-like domain (TGD) and a β-hairpin domain (BHD1), which together associate with an 11-base pair duplex segment flanking the lesion. A second part entails two other β-hairpin domains (BHD2 and BHD3) that bind to a 4-nucleotide segment containing the lesion. In this interaction, the damage-containing base pairs are dislocated from the duplex inducing a flipped-out configuration, which we refer to as the “open” conformation. The BHD2–BHD3 domains embrace the nucleotides on the undamaged strand but not the damaged ones. Furthermore, a long β-hairpin finger protruding from BHD3 is inserted into the DNA, stabilizing the gap created from the flipped-out nucleotides.

In summary, biochemical and structural analyses revealed that the exquisite substrate versatility of yeast Rad4 and its human homolog XPC is achieved by an entirely indirect readout strategy that senses mis- or unpaired bases opposite to a bulky lesion in DNA duplexes. This unprecedented mechanism defies the traditional “fitting glove” [51] or “fitting shoe” strategy [52] whereby lesion recognition takes place through close interactions between a dedicated protein pocket and damaged nucleotide moieties, as shown for many DNA glycosylases participating in base-excision repair (BER). In GG-NER, DNA lesions are instead located by first detecting thermodynamic destabilizations inducing a local single-stranded character. The advantage of this indirect strategy by XPC/Rad4 is that the range of DNA damages sensed for further processing is greatly broadened.

## Interactome of the XPC sensor

In addition to being involved in the association with DNA, the TGD domain is also required for the interaction between Rad4 and Rad23 [50], or between the respective human homologs XPC and RAD23B (see Fig. 2a). Part of the human TGD also interacts with XPA protein [53]. Another partner, known as DDB2 (for *D*amaged *D*NA-*B*inding 2; encoded by the *XPE* gene) appeared more recently in evolution and does not exist in lower eukaryotes like yeast. However, a transient interaction between DDB2 and XPC is pivotal for the processing of CPDs in mammals and the corresponding interaction domain has been mapped to the TGD and BHD1 domains [33]. Besides these central DNA-, RAD23B-, XPA- and DDB2-binding regions, residues 847–863 in the carboxy terminus of XPC form an α-helix that binds tightly to CETN2 [27, 54]. Residues 816-940 in this carboxy-terminal region as well as a portion of the amino-terminal region around residue 334 associate with two distinct subunits (p62 and XPB) of the TFIIH complex [55–57]. In addition, XPC protein interacts with different DNA glycosylases and the Oct4-Sox2 activator of pluripotency (see below). Recently, a high-throughput two-hybrid screen revealed 49 additional potential interactors with roles in DNA synthesis, proteolysis, post-translational modification including the OTU deubiquitinase 4 (OTUD4), transcription, signal transduction and metabolism. However, so far only the association with OTUD4 has been validated by immunoprecipitation [58]. There is also a biochemically proven interaction between human XPC and the USP7 deubiquitinase (for Ubiquitin-Specific-processing Protease 7) [59].

## Search for bulky DNA lesions in the genome

The next challenging question is how the XPC complex scans the genome and succeeds in finding rare lesions with disrupted base pairs within 6.4 billion base pairs of native DNA. Fluorescence-based imaging methods provided a real-time strategy to track the mobility of repair proteins at work in their physiologic milieu in living cells. One main application, fluorescence recovery after photobleaching (FRAP), showed that the movement of the XPC complex is slower than expected from its predicted diffusion rate. This low mobility indicated that the initial complex, unlike the downstream GG-NER factors, does not freely diffuse across the nucleoplasm but associates with native DNA in chromatin while searching for sites of base pair destabilization [60, 61].

The mechanism of DNA damage search was investigated by site-directed mutagenesis of a short β-turn subdomain situated at the transition between the BHD2 and BHD3 domains of human XPC (see Fig. 2a). This particular study focused on a DNA-repulsive glutamic acid residue at codon 755 that is evolutionary conserved and located between two amino acids that make contacts with DNA in the co-crystal of the Rad4 homolog. Conversion of this negatively charged glutamic acid, which clashes with the negatively charged deoxyribose-phosphate backbone of DNA, to the positively charged lysine increased the binding of XPC protein to the double helix. It was noted by FRAP that this charge inversion is sufficient to increase the residence time of XPC on native DNA and, accordingly, decrease its ability to freely move across chromatin. This charge inversion also reduces GG-NER efficiency. The DNA-repulsive residue in this β-turn motif is, therefore, key to efficient genome surveillance, as it prevents XPC from residing too long at any given native DNA site [62].

The importance of preventing a prolonged residence of XPC during its damage search process became evident from recent structural studies of Rad4 bound to native DNA. A co-crystal of Rad4 with undamaged DNA was captured by covalently tethering a TGD residue to duplex DNA [63]. The resulting structure showed that immobilized Rad4 is able to flip-out undamaged nucleotides exactly as observed before on damaged DNA without tethering [50]. This finding demonstrates that, by allowing a prolonged residence on DNA, Rad4 would flip-out even thermodynamically stable nucleotide pairs. This conclusion in turn implies that the binding of XPC to lesions is accomplished not by differences in the most stable, DNA-bound structures between damaged and undamaged DNA (since there is no difference), but by the kinetic probability difference in flipping-out nucleotides before the protein diffuses away. In temperature-jump perturbation spectroscopy experiments, the Rad4-induced DNA opening took ~7 ms at base pair-destabilized target sites, but the same process is orders of magnitude slower on native base pairs [63]. Compared with the sub-millisecond residence time of Rad4 on undamaged DNA, this opening time is too long to result in proficient interactions. Such a kinetic gating mechanism excludes the opening of native DNA while selectively opening damaged sites exhibiting ruptured base pairs.

That the just described “interrogate-and-open” process also takes place in the genome of human cells had been tested using fluorescently labeled XPC truncates. The differential redistribution of truncation products to UV lesion spots revealed that BHD1 and BHD2, together with the β-turn subdomain, are sufficient to interrogate the DNA double helix for the presence of non-hydrogen-bonded bases. To further mature into an open and stable recruitment platform, XPC protein additionally needs the BHD3 domain, which promotes insertion into the double helix of its β-hairpin finger [62]. With this hairpin insertion, the sensor is stably anchored onto the opened DNA duplex displaying two fully flipped-out nucleotides, which allow for the recruitment of the TFIIH complex [64, 65]. To summarize, XPC quickly scans the double helix for base pair integrity before undergoing extensive interactions at destabilized targets to ultimately form the open conformation. This interrogate-and-open process enhances the efficiency of lesion recognition by obviating the futile flipping-out of undamaged base pairs present in large excess.

## Demarcation of bulky DNA lesions for NER activity

It is now clear that XPC does not act as a canonical DNA damage reader but rather as a thermodynamic sensor of ruptured base pairs without making contacts with chemically modified residues. The true role of XPC is to start DNA damage recognition by recruiting the XPD helicase as part of the TFIIH complex [66, 67]. In detail, site-directed mutagenesis of the β-turn and BHD3 regions showed that XPC protein projects the conserved residues Asn754, Phe756 and Phe797 to encircle one non-hydrogen-bonded nucleotide in the undamaged strand opposite to bulky lesions. Extrusion of the adjacent undamaged nucleotide is induced by interactions with Phe797 and Phe799 upon the β-hairpin insertion. The significance of this double flipping-out was tested by complementing XP-C fibroblasts with expression constructs coding for wild-type or mutated XPC protein. Immunochemical analyses showed that a substitution of Phe799 to alanine was sufficient to inhibit recruitment of the TFIIH complex to spots of UV lesions, thus demonstrating that the flipping-out of two nucleotides from the undamaged strand is a crucial prerequisite for TFIIH loading [68].

**Fig. 3:**
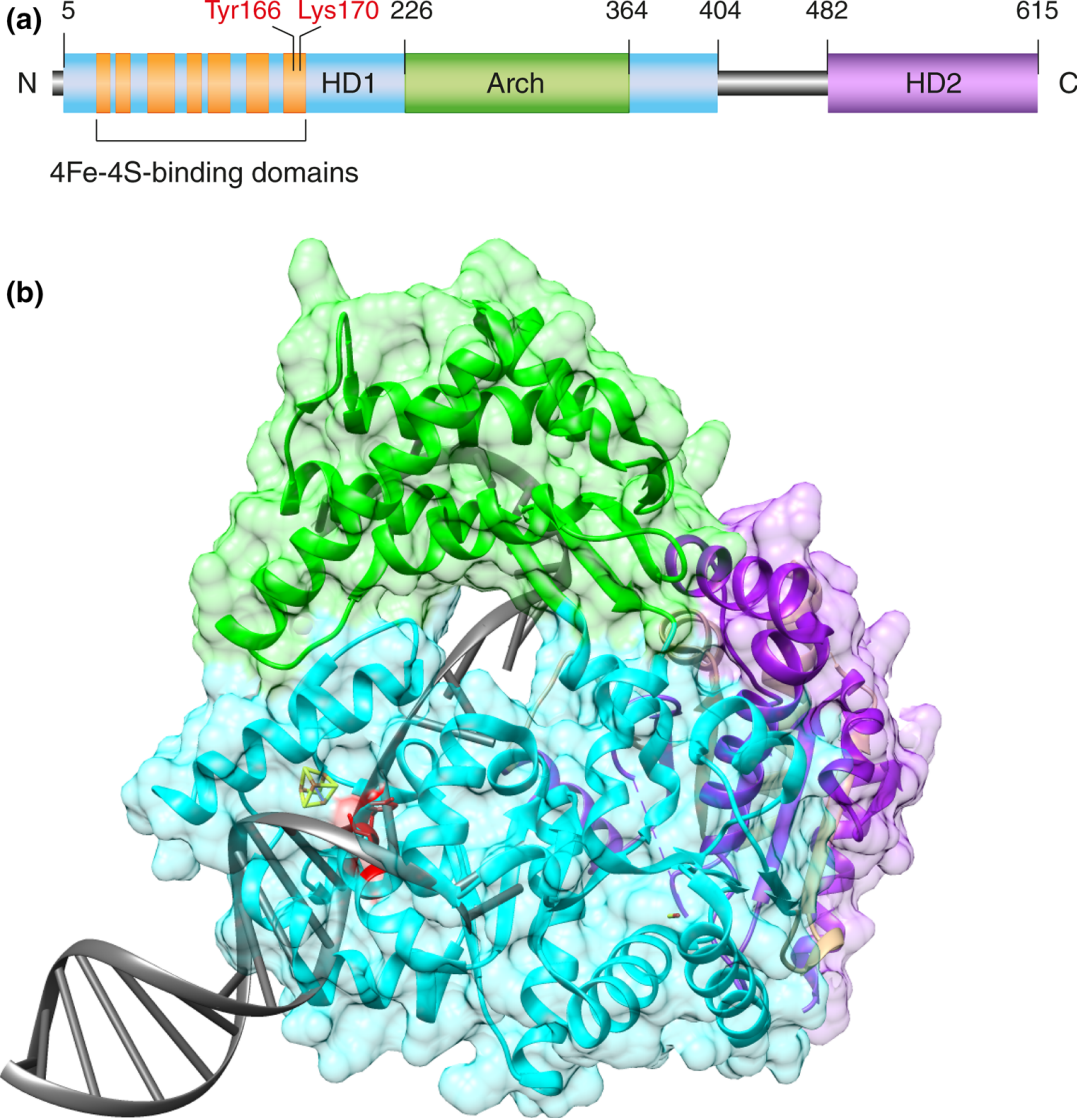
Recognition of bulky DNA lesions by the XPD helicase. **a** Domain map of XPD protein from *Thermoplasma acidophilum* highlighting the helicase motor domains (HD1 and HD2), the Arch domain and the 4Fe–4S cluster. The evolutionary conserved amino acids Tyr166 and Lys170 (Tyr192 and Arg196 in the human homolog) are responsible for the recognition of damaged bases. **b** Ribbon diagram of the XPD helicase from *T. acidophilum* [152] modeled in complex with DNA to illustrate how one strand is thought to penetrate the central protein hole during the unwinding process. *Green* Arch domain; *light blue* HD1; *purple* HD2; *red* amino acids Tyr166 and Lys170; *gray* DNA. The residues Tyr166 and Lys170 (Tyr192 and Arg196 in the human homolog) are located in a strategic position near the central hole where they immobilize damaged bases just before they enter the protein tunnel [72]. The figure was prepared with the Chimera extensible molecular modeling system, using PDB accession codes 4A15 for the XPD [152] and 2P6R for the DNA [153]

The XPD subunit of TFIIH displays a 5′-3′ helicase activity that provides a directional tracking engine for the scanning of individual DNA strands. Major structural and functional features of this helicase were deduced from the analysis of homologous proteins in archaeal organisms [69–71]. Their crystal structure revealed that XPD consists of two helicase motor domains (HD1 and HD2), an iron–sulfur cluster (4Fe–4S) and an auxiliary Arch domain (Fig. 3a). During enzyme translocation driven by ATP hydrolysis, the 4Fe–4S cluster and Arch domain are thought to cooperate in separating the complementary strands of duplex DNA, in a way that one strand enters a narrow hole of the enzyme and then moves along an internal channel, while the opposing strand is displaced to the backside of the protein (Fig. 3b).

Another archaeal homolog from *Ferroplasma acidarmanus* was used to analyze how XPD responds to a CPD lesion located either in the translocated strand entering the helicase hole or in the displaced backside strand. Biochemical assays showed that the helicase activity was blocked by a CPD and that this stalling of the helicase gives rise to a long-lived recognition complex that demarcates the lesion in the translocated but not in the displaced strand [67]. To understand this XPD-dependent scanning mechanism, amino acids in an evolutionary conserved nucleotide-binding surface near the entrance of the narrow hole were targeted by site-directed mutagenesis. Two of the resulting mutants retain ATPase and helicase activity, but in contrast to the wild-type control, are not arrested by a CPD while tracking along DNA. When the consequences of these mutations were tested in living cells, the two mutant XPD proteins failed to induce long-lived demarcation complexes at UV lesion spots and conferred defective GG-NER activity [72]. These reports prove that XPD is the *de facto* DNA damage recognition subunit in the NER pathway by trapping offending bases in a pocket of the enzyme surface just before they enter the narrow helicase hole (Fig. 3b).

## Recruitment of further DNA repair pathways by the XPC sensor

In DNA-binding assays, the selectivity of the XPC sensor extends from bulky DNA lesions to certain smaller or “non-bulky” base modifications, including for example 8-oxo-guanine or methyl-formamidopyrimidine moieties, that are typical substrates of the BER pathway [73]. Accordingly, XPC protein is readily recruited to stripes of 8-oxo-guanines generated in the cell nuclei by low-energy irradiation with a 405-nm laser in the presence of a photosensitizing agent [74]. Other reports demonstrated that the XPC complex stimulates the activity of at least four distinct DNA glycosylases, which initiate BER reactions by cleaving *N*-glycosylic bonds from the deoxyribose-phosphate backbone, i.e., methylpurine-DNA glycosylase (MPG; [75]), thymine-DNA glycosylase (TDG; [76]), 8-oxo-guanine-DNA glycosylase 1 (OGG1; [77, 78]) and single-strand-selective monofunctional uracil-DNA glycosylase 1 (SMUG1; [76]). Moreover, mouse and human cells lacking functional XPC are hypersensitive to the cytotoxic effects of oxidative agents and also display an increased sensitivity to etoposide, a topoisomerase II inhibitor that causes DNA breaks [79]. These different lines of evidence indicate that XPC might use its affinity for destabilized base pairs to serve not only as the initiator of GG-NER activity but also as a more general platform for the loading of multiple repair pathways, including BER and double-strand break repair systems, to damaged DNA carrying compound lesions.

## XPC functions outside DNA repair

TFIIH was the first example of a functional link between the NER system and transcription. Indeed, the TFIIH complex was originally characterized as a basal transcription factor [80, 81] and the discovery that it is also an NER component came later when it was found that XPB and XPD, known to participate in DNA repair, represent ATPase and DNA helicase subunits of this multifunctional enzyme [82]. A second link between transcription and DNA repair was evidenced by the TC-NER sub-pathway, in which DNA damage encountered during transcription is removed through the action of many NER factors that are also involved in the GG-NER process. Although previously believed to act only as damage sensor in the GG-NER pathway, XPC was found to support transcription independently of its DNA repair function. Chromatin immunoprecipitation analyses indicated that XPC protein and, sequentially, downstream NER factors (XPA, RPA, XPG and XPF-ERCC1) home in on the promoter region of nuclear receptor-induced genes [83, 84]. Transcription inhibitors abolish this recruitment of GG-NER factors to active promoters but not to sites of DNA damage, demonstrating that their engagement with gene promoters is functionally distinct from the role in DNA repair. Cell lines with mutated XPC or XPA show reduced levels of mRNA expression from nuclear receptor-activated genes, implying that GG-NER factors optimize the efficiency of transcription. How exactly they co-activate the transcription machinery is not yet clear, but it has been observed that the presence of GG-NER factors in promoters is necessary to orchestrate a more permissive chromatin environment characterized by histone modification changes like H3K4 methylation, H3K9 de-methylation and H3K9 acetylation. One attractive hypothesis is, therefore, that XPC protein and accompanying NER factors exert a non-repair function by remodeling the epigenetic landscape to favor transcription [83]. Along this concept, it is tempting to propose that promoter occupancy by the GG-NER system may serve to install a more accessible chromatin environment regardless of whether the fate of the DNA substrate is to be repaired (in response to DNA damage) or to be transcribed (in response to promoter activation). That the GG-NER system may assemble in the absence of DNA lesions was confirmed by targeting XPC protein to undamaged genomic sites using a high-affinity lactose operator/repressor tethering system [85].

Yet another non-repair function of XPC emerged from the search for transcriptional co-factors that potentiate the Oct4- and Sox2-dependent expression of the *Nanog* pluripotency gene, which is needed for self-renewal of embryonic stem cells as well as for the reacquisition of stem cell-like properties. Using a defined in vitro transcription system, the XPC complex was identified as a co-activator of *Nanog* expression that interacts directly with the Oct4/Sox2 dimer [86]. This unexpected co-activator role was further tested by RNA interference of XPC, RAD23B and CETN2 expression in mouse embryonic stem cells. Down-regulation of the trimeric XPC complex triggered stem cell apoptosis, thus supporting the notion that this factor promotes pluripotency and self-renewal. Moreover, depletion of XPC or RAD23B compromised the induction of pluripotent stem cells from differentiated fibroblasts [86]. Notably, the XPC complex was still capable of co-activating *Nanog* transcription even if XPC contained a mutation (Trp690Ser) that abrogates binding to DNA or a truncation at position 813 that abrogates its interaction with TFIIH ([87]; see domain map in Fig. 2a). Another study even showed that the entire carboxy-terminal region of XPC is dispensable for the transcriptional activity of Oct4-Sox2. In this case, the *XPC* gene of mouse embryonic stem cells was down sized to the first 8 exons, which eliminates a large portion of the coding sequence, from residue 326 to the C terminus, but without compromising pluripotency [88]. However, the expression and stability of the expected amino-terminal XPC fragment of 325 amino acids had not been confirmed. Also, it is not yet possible to reconcile the finding that the XPC complex adopts a role during transcription, in both stem and somatic cells, with the fact that mice lacking the *XPC* gene show no overt developmental defects [89].

## Control of *XPC* expression and cellular localization

In view of its diverse actions as a DNA quality sensor that interrogates the native double helix, permanently scrutinizing base pair integrity, associates with multiple DNA repair systems and also carries out non-repair functions in transcription, it is not at all surprising to note that the cellular level, localization and activity of XPC protein must be kept under tight control. Circumstantial evidence suggests that XPC protein cannot exist in nuclei at high steady-state levels and, therefore, its expression and intracellular concentration must be tuned in accordance to the needs imposed by DNA damage or ongoing transcription. For example, even low expression of the yeast homolog Rad4 interferes with cell growth in *Escherichia coli* [90, 91] and, similarly, microinjection of complementary DNA coding for XPC and RAD23B proteins into human fibroblasts led to cytotoxicity [25]. Finally, it was shown that a faulty regulation of XPC homeostasis causes excessive chromosomal aberrations following UV exposure [92].

Under steady-state conditions in the absence of genotoxic stress, transcription of the *XPC* gene is down regulated by the E2F4-p130 repressor [93]. This transcriptional inhibition is relieved, on the one hand, by Sirtuin 1 (SIRT1), an NAD-dependent deacetylase that induces *XPC* expression by preventing nuclear localization of the E2F4-p130 repressor [94]. The transcriptional inhibition by E2F4-p130 is also relieved by the tumor suppressor ARF (for *A*lternative *R*eading *F*rame), which diminishes the binding of E2F4-p130 to regulatory sequences in the *XPC* gene promoter [95]. In response to UV light, ionizing radiation or alkylating agents, the *XPC* gene is induced by the tumor suppressor p53 [96, 97]. Using chromatin immunoprecipitation assays, a functional p53 binding sequence was identified within the *XPC* gene in an unusual location at the translational start site [98, 99]. Based on the finding that BRCA1 represents another positive transcriptional regulator of the human *XPC* gene, a sequential scenario has been proposed for an involvement of *XPC* in the progression of breast or ovarian cancer, where the loss of BRCA1 restricts the initiation of GG-NER activity by XPC protein and, therefore, causes an accumulation of DNA damage and mutations in the *p53* gene, which in turn leads to an even more pronounced GG-NER defect and genome instability [100].

The intracellular localization of XPC protein is influenced by a DNA damage-sensitive cytoplasmic–nuclear shuttling system. Under unchallenged conditions, XPC continuously shuttles between the cytoplasm and the nucleus driven by a balanced effect of nuclear localization and nuclear export signals in its amino acid sequence. Upon genotoxic stress for example by inflicting UV radiation, there is a shift in this cytoplasmic–nuclear balance towards higher XPC concentrations in the nucleus [25, 60]. The molecular mechanism underlying this nuclear retention in response to DNA damage is not yet understood, but polypeptide modifiers like ubiquitin or SUMO (for *S*mall *U*biquitin-like *MO*difier) have been implicated in the spatiotemporal regulation of XPC protein (see below). It is likely that increased nuclear XPC levels, achieved by enhanced expression as well as increased nuclear retention and reduced degradation, are necessary to optimize the detection of those lesions that are refractory to recognition or less accessible in densely condensed chromatin.

## Support for the XPC sensor by the DDB2 detector

Exposure to UV radiation induces CPDs and 6-4PPs in a ratio of 3:1. These pyrimidine dimers differ in their biophysical properties and genomic distribution: CPDs cause relatively minor base pair destabilizations in duplex DNA compared to 6-4PPs [101–103]. Additionally, CPDs arise evenly across chromatin, whereas 6-4PPs are formed primarily in linker DNA rather than in nucleosome cores [104–106]. Because CPDs are removed at slower rates than 6-4PPs, they display a higher mutagenic potential and are responsible for most adverse effects of UV radiation ranging from sunburns to skin aging and cancer [107, 108].

**Fig. 4:**
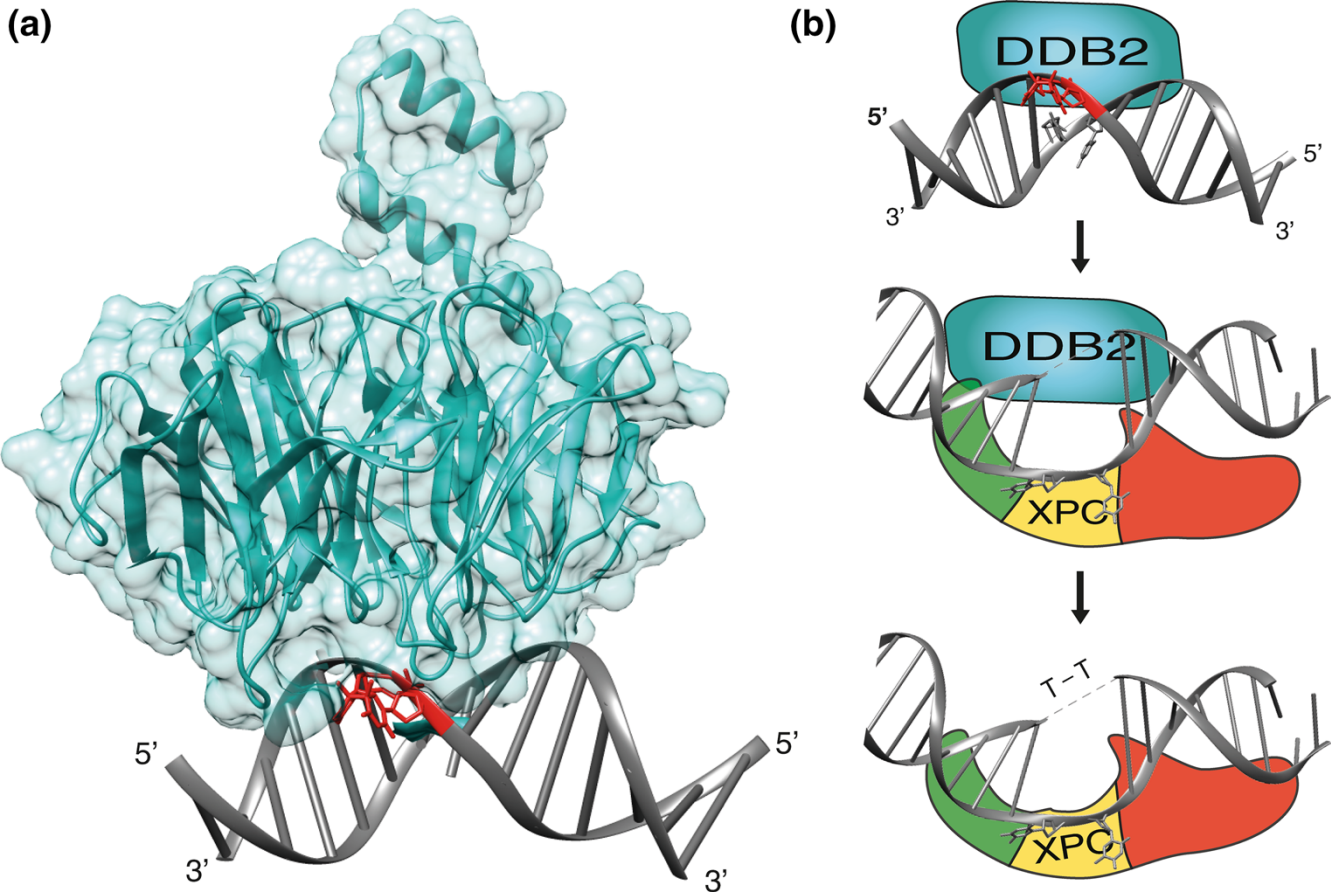
Assistance by the DDB2 damage detector. **a** Ribbon representation of DDB2 from zebrafish in complex with double-stranded DNA containing a CPD lesion [117]. *Blue* DDB2: *gray* DNA; *red* CPD. The figure was prepared with the Chimera extensible molecular modeling system, using PDB accession code 4A09. **b** Recognition of a CPD by the DDB2 damage detector and handover of the lesion to the XPC partner. Binding of DDB2 to the UV lesion in DNA triggers an interaction with the BHD1 fold of XPC protein. This transient association of DDB2 with XPC at lesion sites facilitates insertion of the long β-hairpin “finger” of XPC into the DNA duplex, followed by the release of DDB2 [33]. A direct substrate handover from DDB2 to XPC is required for the excision of CPDs that, on their own, induce minimal base pair disruption and, hence, are not recognizable by XPC alone. *Light* *blue* DDB2; *Green*, *yellow* and *red* BHD1, BHD2 and BHD3 folds of XPC, respectively

Despite being the repair initiator for all bulky DNA lesions including CPDs, purified XPC protein does not bind CPDs with any measurable selectivity [43, 47, 109, 110]. This lack of specificity for CPDs is compensated by DDB2 (the factor mutated in XP-E patients; [111, 112]) whose transcription is also induced by the p53 and BRCA1 tumor suppressors, as seen for the *XPC* gene [113, 114]. Unlike XPC, which functions as a general sensor of helix disruptions independently of the nature of the offending lesion, DDB2 is specialized on the recognition of CPDs and 6-4PPs [115]. Crystal structures of DDB2 revealed a binding pocket, in the center of its β-propeller architecture, that is tailored towards high-affinity binding of CPDs and 6-4PPs while excluding larger base adducts (Fig. 4a; [116–118]).

The absence of a functional DDB2 protein in XP-E cells nearly abolishes the excision of CPDs although the repair of 6-4PPs is only slightly reduced [113, 119]. A widely accepted model is that DDB2 recognizes the CPDs and then delivers them to XPC for initiation of GG-NER activity [115, 120, 121]. However, the precise handover mechanism remained elusive for a long time because, in biochemical assays, purified DDB2 and XPC proteins compete directly for UV lesions and it was not possible to isolate stable intermediates where these two factors bind together to one damaged DNA site [122]. An explanation for this failure to isolate ternary DDB2-XPC-DNA intermediates came from the individual co-crystals. Each structure showed a DNA kink at the lesion site, but the kinks were in diametrically opposite directions when compared with each other. Moreover, both DDB2 and XPC insert a β-hairpin finger into the double helix, such that one would clash with the concomitant binding of the other [50, 116, 117].

The mechanism of substrate handover from DDB2 to XPC was eventually investigated using methods that detect short-lived interactions in the chromatin environment, including in situ domain mapping at spots of UV lesions and FRAP on local damage (FRAP-LD), combined with biochemical assays using isolated XPC domains [33]. These studies demonstrated that XPC lends two of its DNA-binding domains (TGD and BHD1) to interact transiently with DDB2 bound to a CPD or 6-4PP lesion. This short-lived intermediate at the site of damage facilitates the insertion of the β-hairpin of BHD3 into the DNA duplex, thereby pulling DDB2 away (Fig. 4b). It is important to point out that the β-hairpin insertion by XPC involves an energetic cost as it occurs by local breakage of stacking interactions and hydrogen bonds between the bases. Though 6-4PPs facilitate this insertion by lowering the melting temperature at the site of damage, XPC protein depends on DDB2 to interact productively with CPD sites, thus explaining the defect of XP-E cells in repairing CPD lesions.

## Post-translational modification of XPC with polypeptide modifiers

In addition to serving as a direct UV lesion detector, the DDB2 protein exists in complex with the adaptor protein DDB1 that recruits the cullin 4A (CUL4A) scaffold and the RING finger protein ROC1 (for Regulator of Cullins 1), which together form the CRL4^DDB2^ ubiquitin ligase. By mediating the covalent attachment of one or more 8-kDa ubiquitin moieties to target proteins [11], this cullin-type ligase provides an additional layer in the fine-tuning of GG-NER activity. Under unchallenged conditions, the CRL4^DDB2^ ubiquitin ligase is maintained in an inactive state by a further association with the COP9 signalosome, a multi-subunit regulatory protease [117]. Upon detection of UV lesions by DDB2, the COP9 signalosome is released, allowing for the modification of CUL4A with the ubiquitin-like modifier NEDD8, which in turn activates the ubiquitin ligase to modify substrates located within approximately 100 Å, generating Lys48-linked ubiquitin chains [116]. The main ubiquitination targets include histones H2A, H3 and H4 as well as DDB2 itself and its interaction partner XPC [12, 123–126].

Upon UV exposure, the CRL4^DDB2^-mediated ubiquitination of histones is thought to help opening chromatin and facilitate the access of repair systems to damaged DNA [125], but this hypothesis is challenged by the finding that CUL4A conditional-knockout mice show higher rather than reduced GG-NER activity [127]. It is, therefore, possible that the CRL4^DDB2^ ligase may have a more specific regulatory role with functional nuances depending on the organism (human or mouse), cell type or genetic background. There is, however, concordance on the finding that the auto-ubiquitination of DDB2 not only abrogates its DNA-binding ability but also triggers a rapid degradation by the 26S proteasome [12]. The same CRL4^DDB2^ ligase ubiquitinates XPC but, in this case, XPC retains its DNA-binding activity and is partially protected from proteasomal destruction (see below). XPC protein is additionally modified with Lys63-linked ubiquitin chains by a separate ligase known as RNF111/Arkadia [128]. This further ubiquitination is contingent on a prior UV-dependent modification of XPC with SUMO [129]. Thus, GG-NER activity in response to UV damage is controlled by a variety of polypeptide modifiers, including SUMO, Lys48- and Lys63-linked ubiquitin chains, which decorate XPC protein at 15 or more distinct modification sites. Interestingly, down-regulation of CRL4^DDB2^ or Arkadia have opposite effects by inhibiting or stimulating, respectively, the accumulation of XPC at UV damage spots, indicating that Lys48-linked ubiquitin chains (produced by CRL4^DDB2^) and Lys63-linked counterparts (produced by Arkadia) exhibit diverging modulating roles [128, 130, 131].

## Control of XPC dynamics in the chromatin of UV-irradiated cells

The packaging of genomic DNA is a compromise between two opposite needs: the DNA must be compacted to fit into the nucleus but still remain accessible to biological processes including DNA repair. To accomplish this dual requirement, DNA assembles with histones to generate a condensed array structure whose basic unit is the nucleosome (reviewed by [132, 133]). Each nucleosome repeat consists of a core particle, where 147 base pairs of the DNA duplex are wrapped around an octamer of core histones (two each of H2A, H2B, H3 and H4), separated by linker DNA of variable lengths. In higher eukaryotes, additional levels of packaging are achieved by interactions of histone H1 with linker DNA.

It is fundamentally important to view the regulatory role of polypeptide modifiers during repair within this native chromatin context. A conceptually new contribution to understanding the function of CRL4^DDB2^-mediated ubiquitination came from the enzymatic dissection of chromatin by micrococcal nuclease (MNase). This enzyme digests DNA in the accessible linker more easily than that in nucleosome cores. Therefore, MNase treatments generate a soluble supernatant containing non-histone proteins that, before digestion, were associated with inter-nucleosomal linkers (amounting to ~35 % of total genomic DNA). Even at saturating enzyme levels, however, MNase digestions leave behind the vast majority of nucleosome core particles (amounting to ~60 % of total DNA) in the form of a densely packed and insoluble nucleoprotein fraction [134].

Two previous findings led us to predict that the CRL4^DDB2^ activity in response to UV irradiation is not uniformly distributed across this nucleosome landscape consisting of core particles divided by linker segments. First, DNA-binding assays demonstrated that DDB2, the UV lesion-binding subunit of CRL4^DDB2^, associates with a 15-fold higher affinity with 6-4PPs (*K*_a_ = 1.5 × 10^9^ M^−1^) compared to CPDs (*K*_a_ = 1 × 10^8^ M^−1^) [110, 135]. Second, 6-4PPs are formed mainly in linker DNA [104, 106]. For these reasons, it was not surprising to observe that, immediately upon UV irradiation, DDB2 associates predominantly with MNase-hypersensitive, highly accessible inter-nucleosomal sites [33]. On the other hand, it was generally thought that XPC on its own is unable to interact with DNA wrapped onto nucleosome cores [44] but, against this prevailing concept, the MNase probing revealed that XPC protein associates rather evenly with MNase-resistant, densely packed nucleosomes and MNase-sensitive inter-nucleosomal DNA. Upon UV irradiation, the binding of XPC to MNase-resistant core particles is further enhanced [33] and this finding is supported by cell imaging studies indicating that XPC is recruited to the condensed chromatin of interphase nuclei [136] and to condensed mitotic chromosomes [60].

**Fig. 5:**
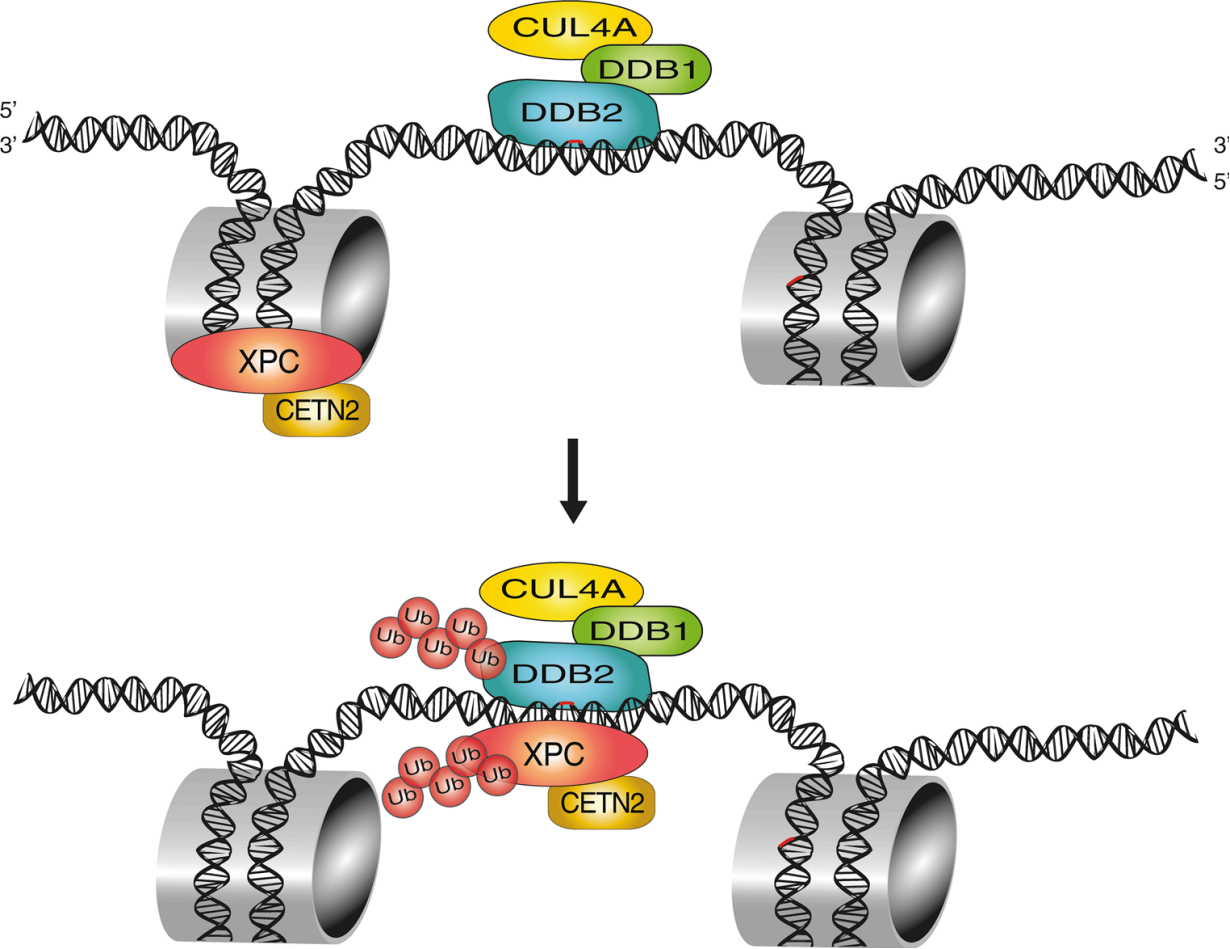
Spatiotemporal control of XPC distribution in chromatin. The cullin-type CRL4^DDB2^ ligase complex prioritizes the excision of UV lesions located in highly accessible chromatin sites. A preferential binding of the damage detector DDB2 to UV lesions in inter-nucleosomal DNA leads to the recruitment and ubiquitination of the XPC partner. This conjugation with polypeptide modifiers promotes the temporary retention of XPC at inter-nucleosomal sites, thus suppressing its constitutive association with nucleosome core particles. This transient ubiquitin code on XPC is necessary for the fast excision of UV lesions from inter-nucleosomal DNA. Thereafter, DDB2 is progressively degraded whereas XPC is de-ubiquitinated to allow for the recognition of UV lesions in nucleosome core particles [33]

In accordance with the preference of DDB2 for UV lesions located in inter-nucleosomal DNA, the entire CRL4^DDB2^ ubiquitin ligase complex is recruited mainly to these accessible sites after UV irradiation. The consequence of this partitioning is that essentially only the fraction of XPC bound to inter-nucleosomal DNA is ubiquitinated whereas XPC bound to condensed core particles avoids ubiquitination [33]. The role of CRL4^DDB2^ was then challenged using the following strategies: down-regulation of either DDB2 or CUL4A using RNA interference, depletion of nuclear ubiquitin using the proteasome inhibitor MG132 or blockage of ubiquitination using the small-molecule E1 inhibitor PYR-41 [137]. Alternatively, the ubiquitination of XPC was suppressed in mouse cells expressing a temperature-sensitive E1 mutant or an XPC-GFP fusion protein that is refractory to ubiquitination. In all these cases, the XPC molecules remained without ubiquitin modifications and were nearly completely relocated to the fraction of packed nucleosome core particles [33]. We, thus, concluded that the CRL4^DDB2^-mediated ubiquitination of XPC serves to retain XPC at inter-nucleosomal sites, representing DNA repair hotspots for the efficient recruitment of downstream NER factors and fast UV lesion excision (Fig. 5). In the absence of CRL4^DDB2^ activity, more XPC binds to CPDs located within nucleosome core particles that represent a less permissive chromatin environment with poor recruitment of downstream GG-NER factors and slow excision of UV lesions. The default-mode association of XPC with core particles, counteracted by CRL4^DDB2^-mediated ubiquitination, contradicts a long-held notion derived from in vitro reconstitution experiments that nucleosomes pose a barrier to recognition of UV lesions by XPC [44, 138]. In summary, these studies showed that the CRL4^DDB2^-mediated ubiquitination serves to establish a distinctive spatiotemporal distribution of the XPC sensor thereby optimizing the recruitment of downstream NER factors in mammalian chromatin (Fig. 5).

## Narrow time window of XPC regulation by CRL4^DDB2^

The nearly instantaneous auto-ubiquitination of DDB2 by CRL4^DDB2^, and ensuing proteolytic degradation of DDB2, translates to an automatic time machine that restricts the ubiquitin ligase activity and its regulatory influence on the XPC partner, to a window of only few hours after UV irradiation. Due to DDB2 breakdown, the degree of XPC ubiquitination diminishes progressively and, as a consequence, this repair initiator relocates from inter-nucleosomal DNA to not yet repaired UV lesions, mainly CPDs, in nucleosome core particles [33]. The time window of this CRL4^DDB2^ action may be prolonged by simultaneous post-translational modifications with poly(ADP-ribose) (PAR) occurring within seconds after UV exposure. Down-regulation of the enzyme poly(ADP-ribose) polymerase 1 (PARP1), by treatment with a small-molecule inhibitor or by RNA interference, reduced the PAR accumulation at UV damage spots and inhibited the excision of CPDs [139, 140]. One report proposes a scenario where PARP1 modifies DDB2 and thereby competes with concurrent ubiquitination, which results in enhanced stability and chromatin retention of the DDB2 subunit [139]. In another study, opposite effects were observed because PARP1 inhibition prevented ubiquitination and removal of DDB2 from chromatin, thus indicating that PARP1 stimulates the DDB2 turnover [140]. Regardless of how exactly PARP1 impinges on the DDB2 half-life, the formation of PAR at sites of UV damage may generate a dynamic scaffold that promotes transient interactions of DDB2 with XPC and facilitates the recruitment of adjuvant factors that stimulate DNA repair like for example the ALC1 (for *A*mplified in *L*iver *C*ancer 1) chromatin remodeler [139] or histone-acetylating enzymes [141, 142].

## Ubiquitin-dependent extraction of DDB2 and XPC from chromatin

Even though the DDB2 damage detector is needed for efficient excision of UV lesions, particularly CPDs, Lys48-linked ubiquitination triggers its degradation within few hours after exposure to UV light [123, 143]. It remained enigmatic why UV radiation induces the degradation of most DDB2 subunits well before excision of CPDs from the genome is completed. The actual scope of this apparently paradoxical breakdown of a DNA lesion detector remained unclear. There are also controversial findings as to whether XPC is partially degraded in response to UV damage [12, 25, 129].

Addressing these questions, it has been demonstrated that both DDB2 and XPC, once modified with Lys48 ubiquitin chains, become a substrate of the ubiquitin-selective p97 segregase, also known as valosin-containing protein (VCP) [92]. Individual p97 subunits assemble to form hexamers that convert ATP hydrolysis into mechanical force, which is used to extract ubiquitinated conjugates from cellular structures [144, 145]. The recognition of ubiquitinated DDB2 and XPC by p97 was demonstrated in situ on UV lesions spots in the nuclei of human cells, and confirmed biochemically by demonstrating that Lys48-ubiquitinated DDB2 and p97 reside in the same multi-protein complex. This recruitment of p97 to ubiquitinated DDB2 and XPC was shown to depend on various adapter proteins known to confer substrate specificity to the p97 segregase [146, 147].

The newly discovered involvement of p97 segregase activity in the GG-NER pathway provided an elegant strategy to test the consequences of an uncontrolled accumulation of DDB2 or XPC in chromatin. For that purpose, p97 activity was down regulated either by RNA interference or, alternatively, by mild overexpression of a dominant-negative mutant, which still binds ubiquitinated proteins but lacks segregase function and, consequently, remains trapped on ubiquitinated substrates [148]. With decreased p97 activity, there was excessive accumulation of both DDB2 and XPC in spots of UV lesions, indicative of an abnormal retention in UV-damaged chromatin, but without any increased recruitment of downstream NER factors like XPB (a component of the TFIIH complex) or ERCC1 (subunit of the ERCC1-XPF endonuclease complex). This down-regulation of p97 inhibited the UV-induced proteolytic clearance of DDB2 and also increased the level of ubiquitinated XPC. Unlike DDB2-ubiquitin conjugates, ubiquitinated XPC is processed in a p97-dependent manner by the USP7 deubiquitinase, thus restoring unmodified protein [59].

Despite their undisputed roles in the initiation of GG-NER activity, abnormally persisting DDB2 and XPC reduce the rate of UV lesion excision. This compromised DNA repair efficiency translates to hypersensitivity to UV radiation as well as enhanced chromosomal aberrations after UV exposure. Importantly, the genome instability observed in UV-irradiated cells after p97 depletion was reversed by concurrent down-regulation of DDB2 or XPC [92]. These findings suggested that the accumulation of either DDB2 or XPC is detrimental and that a tight control of their levels in chromatin is essential for genome stability. If this hypothesis were correct, then excessive expression of one of these factors would be sufficient to cause genome instability. In support of this intriguing concept, we observed that in a background of normal p97 activity, overexpression of wild-type DDB2 but not overexpression of a defective DDB2 mutant, inhibited the excision of UV lesions and enhanced the frequency of chromosomal aberrations after UV exposure. Double overexpression experiments involving both DDB2 and p97 demonstrated that increased levels of chromatin-bound DDB2 compromise genome stability only as long as they exceed the turnover capacity of the p97 segregase. Thus, a strict spatiotemporal control of the chromatin homeostasis of DDB2 and XPC by the p97 segregase is critical for efficient NER activity and a key function of the Lys48-linked ubiquitin modification of DDB2 and XPC is to prime these initial NER players for subsequent release from chromatin [92].

**Fig. 6:**
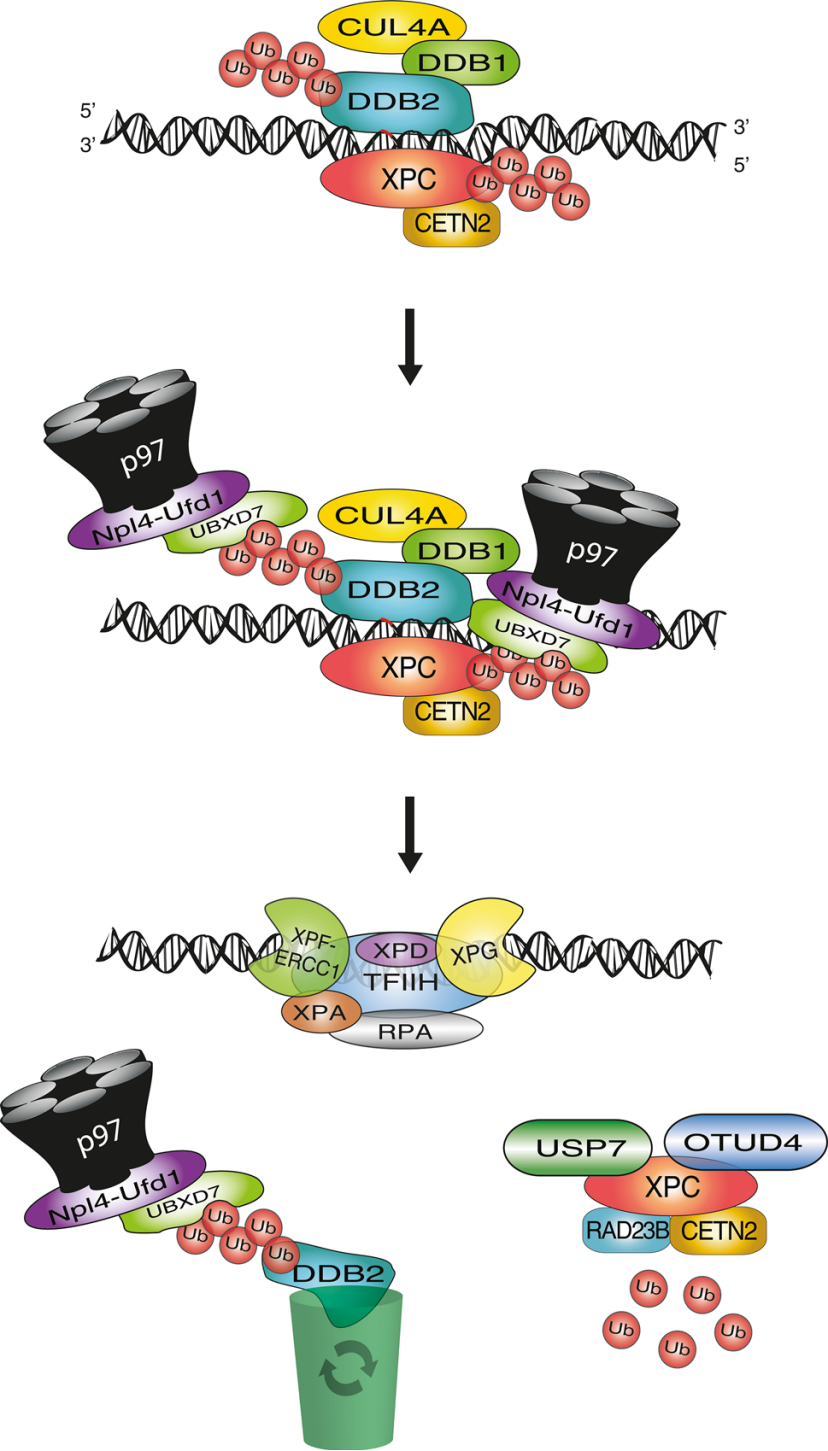
Extraction of the UV detector DDB2 and damage sensor XPC from chromatin. The p97 segregase complex regulates GG-NER activity by removing ubiquitinated DDB2 and XPC from chromatin, thus favoring downstream recognition (by TFIIH) and excision steps (by XPF-ERCC1 and XPG). Ubiquitinated DDB2 is delivered to the proteasome system for degradation, whereas XPC is mostly recycled by de-ubiquitination through the action of USP7 or other de-ubiquitinating enzymes like OTUD4 [58, 59, 92]

The paradigm of DDB2 homeostasis illustrates how both low and high levels of a DNA damage recognition factor impede repair and cause genome instability (Fig. 6). DDB2 stimulates the excision of UV lesions but, if bound to damaged chromatin in excess due to a failure in its extraction or degradation, this same sensor acquires genotoxic properties culminating in chromosomal aberrations. Evidently, DNA damage sensors such as DDB2 and XPC act as a double-edged sword as they trigger a beneficial defense but become unfavorable if allowed to accumulate in chromatin without control by the p97 segregase.

## Conclusion

The XPC complex functions as the general initiator of GG-NER activity by virtue of its ability to sense the presence of unpaired bases in double-stranded DNA and recruit the XPD verifier for subsequent bulky lesion confirmation. The clinical feature of a mutated *XPC* gene in xeroderma pigmentosum (hypersensitivity to UV radiation and skin cancer) highlights the extraordinary importance of this repair-initiating function for the excision of photodimers (CPDs and 6-4PPs) induced by sunlight exposure.

In a wider perspective, life on the planet Earth would not have been possible without the emergence of effective DNA repair mechanisms for the removal of UV photolesions. Indeed, most living organisms exposed at least transiently to sunlight possess a very rapid, highly efficient and safe enzymatic tool for the repair of photolesions in the form of DNA photolyases that, by light-driven catalysis, revert CPDs or 6-4PPs to pyrimidine monomers without excision of bases or any deoxyribose-phosphate residues [149, 150]. Unlike other animals, however, placental mammals are devoid of this simple light-driven DNA repair activity, presumably because they evolved from strictly nocturnal species originating from the Cretaceous era [151]. To finally return to a diurnal life under sunlight, placental mammals needed to “upgrade” their GG-NER pathway that constituted a hazardous backup system for the excision of base lesions refractory to photolyases or similarly innocuous reversal processes. In principle, many potential disadvantages are associated with implementation of the GG-NER system as the sole DNA repair defense against bulky UV lesions in placental mammals. First, CPDs would escape repair because the generic XPC sensor initiating GG-NER activity is not able to detect this most prevalent type of UV lesion. Second, sunlight-exposed skin cells would be faced with the uncoordinated cleavage of their DNA at thousands or more chromosomal sites nearly simultaneously, which would unavoidably threaten genome stability. Third, CPDs arise with a uniform pattern throughout the genome, including highly condensed sites that are poorly accessible and refractory to the assembly of GG-NER complexes.

Fascinating advances of the last decade in the field of GG-NER indicate that these aforementioned problems are countered inter alia by the following strategies. First, the accessory UV damage detector DDB2 attracts the XPC complex to CPDs that would otherwise remain unrecognized. Second, the repair-initiating activity of XPC is spatially regulated. By recruitment of the CRL4^DDB2^ ligase mediating XPC ubiquitination, activity of the GG-NER pathway is in the beginning limited to highly accessible nucleosome-free sites that are easily amenable to the entire set of downstream excision factors, thus protecting more compacted chromatin localizations from accidental incisions that could trigger chromosome fragmentation. Third, the repair-initiating activity of XPC is temporally regulated. Through degradation induced by the CRL4^DDB2^ ubiquitin ligase, the repair-stimulating activity of DDB2 is self-limiting and lasts only few hours after an acute dose of UV damage. Fourth, XPC is able to sense UV lesions within tightly packed nucleosomes and, by a not yet understood epigenetic mechanism affecting the local histone code, generates a more DNA repair-permissive chromatin landscape. This latter mechanism is also employed for chromatin rearrangements occurring during transcriptional reprogramming of cells independently of DNA damage. Finally, rapid extraction of a surplus of ubiquitinated DDB2 and XPC from chromatin ensures optimal GG-NER activity and avoids molecular collisions with other ongoing processes like transcription or DNA replication. Only by adoption of these regulatory circuits during mammalian evolution, it has become possible to deploy the GG-NER pathway as the sole DNA repair system protecting from the mutagenic and carcinogenic effects of UV light.

## Abbreviations

6-4PP: (6-4) Photoproduct
ALC1: Amplified in liver cancer 1
ARF: Alternative reading frame
BER: Base excision repair
BHD: β-Hairpin domain
BRCA1/2: Breast cancer 1/2
CETN2: Centrin 2
CPD: Cyclobutane pyrimidine dimer
CUL4A: Cullin 4A
DDB: Damaged DNA-binding
ERCC1: Excision repair cross-complementing 1
GG-NER: Global-genome nucleotide excision repair
MPG: Methylpurine-DNA glycosylase
NER: Nucleotide excision repair
OGG1: 8-Oxo-guanine-DNA glycosylase
OTUD4: OTU deubiquitinase 4
PAR: Poly(ADP-ribose)
PARP1: Poly(ADP-ribose) polymerase 1
RAD23B: Human homolog of RAD23, B
RPA: Replication protein A
ROC1: Regulator of cullins 1
SIRT1: Sirtuin 1
SMUG1: Single-strand-selective monofunctional uracil-DNA glycosylase 1
SUMO: Small ubiquitin-like modifier
TC-NER: Transcription-coupled nucleotide excision repair
TDG: Thymine-DNA glycosylase
TFIIH: Transcription factor IIH
TGD: Transglutaminase-like domain
USP7: Ubiquitin-specific processing protease 7
UV: Ultraviolet
VCP: Valosin-containing protein
XP: Xeroderma pigmentosum
